# Genetic control of the operculum and capsule morphology of *Eucalyptus globulus*

**DOI:** 10.1093/aob/mcac072

**Published:** 2022-06-02

**Authors:** Mariano A Hernández, Jakob B Butler, Hans Ammitzboll, James L Weller, René E Vaillancourt, Brad M Potts

**Affiliations:** School of Natural Sciences, University of Tasmania, Hobart, Tasmania 7001, Australia; ARC Training Centre for Forest Value, University of Tasmania, Hobart, Tasmania 7001, Australia; Instituto Nacional de Tecnología Agropecuaria (INTA), Route 27 - Km 38.3, Bella Vista, Corrientes 3432, Argentina; School of Natural Sciences, University of Tasmania, Hobart, Tasmania 7001, Australia; School of Natural Sciences, University of Tasmania, Hobart, Tasmania 7001, Australia; ARC Training Centre for Forest Value, University of Tasmania, Hobart, Tasmania 7001, Australia; School of Natural Sciences, University of Tasmania, Hobart, Tasmania 7001, Australia; Australian Research Council Centre of Excellence for Plant Success in Nature and Agriculture; School of Natural Sciences, University of Tasmania, Hobart, Tasmania 7001, Australia; ARC Training Centre for Forest Value, University of Tasmania, Hobart, Tasmania 7001, Australia; School of Natural Sciences, University of Tasmania, Hobart, Tasmania 7001, Australia; ARC Training Centre for Forest Value, University of Tasmania, Hobart, Tasmania 7001, Australia

**Keywords:** Beak, calyptra, candidate genes, capsule, *Eucalyptus globulus*, fruit, genetic control, ontogenetic development, opercula, pleiotropy, QTL analysis

## Abstract

**Background and aims:**

The petaline operculum that covers the inner whorls until anthesis and the woody capsule that develops after fertilization are reproductive structures of eucalypts that protect the flower and seeds. Although they are distinct organs, they both develop from flower buds and this common ontogeny suggests shared genetic control. In *Eucalyptus globulus* their morphology is variable and we aimed to identify the quantitative trait loci (QTL) underlying this variation and determine whether there is common genetic control of these ecologically and taxonomically important reproductive structures.

**Methods:**

Samples of opercula and capsules were collected from 206 trees that belong to a large outcrossed F_2_*E. globulus* mapping population. The morphological variation in these structures was characterized by measuring six operculum and five capsule traits. QTL analysis was performed using these data and a linkage map consisting of 480 markers.

**Key results:**

A total of 27 QTL were detected for operculum traits and 28 for capsule traits, with the logarithm of odds ranging from 2.8 to 11.8. There were many co-located QTL associated with operculum or capsule traits, generally reflecting allometric relationships. A key finding was five genomic regions where co-located QTL affected both operculum and capsule morphology, and the overall trend for these QTL was to affect elongation of both organs. Some of these QTL appear to have a significant effect on the phenotype, with the strongest QTL explaining 26.4 % of the variation in operculum shape and 16.4 % in capsule shape. Flower bud measurements suggest the expression of these QTL starts during bud development. Several candidate genes were found associated with the QTL and their putative function is discussed.

**Conclusions:**

Variation in both operculum and capsule traits in *E. globulus* is under strong genetic control. Our results suggest that these reproductive structures share a common genetic pathway during flower bud development.

## INTRODUCTION

The genus *Eucalyptus* has many distinctive morphological features including those of its reproductive structures (Boland *et al.*, 1992; Slee *et al.*, 2020). Flowers of *Eucalyptus* feature an operculum (also referred to as a calyptra) that is derived from the fusion of petals, sepals or both (Florence, 1996; Vasconcelos *et al.*, 2019). This structure is believed to protect flower organs against dehydration and predation until anthesis (Vasconcelos *et al.*, 2017), at which point it is shed to expose the expanded filaments and nectar that attract and reward pollinators (House, 1997). The operculum has considerable implications in the evolutionary history of *Eucalyptus*, as variation in its ontogenesis and morphology is phylogenetically structured and taxonomically significant (Carr and Carr, 1969; Pryor and Knox, 1971; Drinnan and Ladiges, 1989; Ladiges *et al.*, 1995). Similarly significant are the fruiting structures which develop from the fertilized eucalypt flower (Bohte and Drinnan, 2011). Following fertilization, the receptacle swells and eventually dries to become a woody capsule (Brooker and Kleinig, 1999), botanically known as a false fruit as it develops from an inferior and multilocular ovarium fused with the hypanthium (Cremer, 1965). Capsules function to shield the seed from predators (Pryor and Johnson, 1981) and fire (dos Santos *et al.*, 2015; Silva *et al.*, 2016), and in most eucalypts are retained in the tree crown several years after fertilization, providing a major source of propagules for regeneration following environmental disturbances (Yates *et al.*, 1995; Gill, 1997; Ashton, 2000). Much like the operculum, capsules vary widely in morphology and internal structure among eucalypts and can be taxonomically diagnostic (Williams and Brooker, 1997).

The common ontogenetic development of opercula and capsules would argue for possible shared genetic control underlying the development of these reproductive structures. Studies of the genetic control of the morphology of opercula and capsules are scarce and the degree of correlation in the development of these traits is unknown. Within eucalypt species, there is often considerable phenotypic variation of operculum and capsule morphology (Ladiges and Ashton, 1974; Kirkpatrick, 1975; Hopper and Burgman, 1983), which may show geographical clinal variation in the wild (Potts and Reid, 1985; Marginson and Ladiges, 1988; McDonald *et al.*, 2009). However, the extent to which such variation is genetically based is poorly understood. One study of a common garden field trial showed that such population variation in capsule size (based on wet weight) was under significant genetic control (McGowen *et al.*, 2004), while other reports show that opercula and capsules have intermediate size in inter-specific eucalypt hybrids compared to the pure parental species (Pryor, 1957; Stokoe *et al.*, 2001; Meddings *et al.*, 2003), again providing evidence of genetic control. In contrast to *Eucalyptus*, numerous quantitative trait loci (QTL) have been identified for fruit weight, size and shape in several economically important crops (Causse *et al.*, 2002; Barchi *et al.*, 2009; Eduardo *et al.*, 2011), although few studies integrate these findings with QTL underlying flower traits (Frary *et al.*, 2003, 2004). Understanding the extent to which the morphology of opercula and capsules is under genetic control and correlated should provide first insight into the common development of reproductive structures in *Eucalyptus*.

The present study focuses on the Tasmanian blue gum *Eucalyptus globulus* Labill., which is endemic to the island of Tasmania, the Bass Strait islands and the adjacent coastal regions of Victoria in continental Australia (Dutkowski and Potts, 1999). It is one of the most important pulpwood plantation eucalypt species in the world and the eucalypt species for which the genetic control of reproductive traits has been most studied (Potts *et al.*, 2004, 2008). *Eucalyptus globulus* is part of a complex of four taxa (*E. globulus* complex), the cores of which are geographically separated but are clinally linked (Jones *et al.*, 2013). These taxa are mainly separated based on reproductive characteristics, including the number of buds per umbel as well as flower bud and capsule size (Kirkpatrick, 1974). Core *E. globulus* has a single bud per umbel and the largest buds and capsules in the complex (Jordan *et al.*, 1993; Jones *et al.*, 2002). Its perianth is formed by an outer membranous sepaline operculum shed early during bud development and an inner petaline operculum that persists and is shed at anthesis (Weberling, 1989; Jones *et al.*, 2011). The present study focuses only on the cap-like corolline structure or inner petaline operculum (hereafter operculum). This operculum has features which are indistinguishable from those of the bud receptacle that eventually forms the seed-bearing woody capsule after fertilization (e.g. surface texture, waxiness and colour), supporting the idea that both reproductive structures share a component of their genetic control. Phenotypic variation of operculum and capsule shape is known to occur in natural populations of *E. globulus* (Kirkpatrick, 1975), which along with variation in other flower organs (such as style or number of ovules) is suggested to be under genetic control (Suitor *et al.*, 2009).

Given the taxonomic and ecological significance of operculum and capsule morphology in *Eucalyptus*, the present study uses a QTL approach with an inter-provenance F_2_ cross of *E. globulus* to determine whether: (1) operculum and capsule shape and size are under genetic control; (2) traits within each organ (i.e. operculum and capsule) are phenotypically related; and (3) operculum and capsule traits are under common genetic control (i.e. pleiotropically related).

## MATERIALS AND METHODS

### Genetic material and trial description

To perform the experiment, a large F_2_ mapping family of *E. globulus* was used. This F_2_ family has been previously utilized for linkage mapping (Hudson *et al.*, 2012) and several QTL analyses (Hudson *et al.*, 2014; Butler *et al.*, 2016; Ammitzboll *et al.*, 2018). It was generated by crossing two F_1_ individuals, each originated by controlled crossing of dwarf ecotype grandparents (believed to be unrelated) from Wilsons Promontory in Victoria, with two tall ecotype grandparents from King Island in Bass Strait and Taranna in south-east Tasmania, respectively. The F_2_ family was planted at two sites in Tasmania: Geeveston (43°13′8.60″S, 146°53′54.81″E, 382 m a.s.l.) and Boyer (42°46′44.85″S, 147°5′28.06″E, 14 m a.s.l.) in November 2006 and May–July 2007, respectively. A randomized design with trees planted at 4 × 4-m spacing was used at each site.

### Assessment of operculum and capsule traits

Opercula were collected in spring 2010 and capsules were collected during the summers of 2015–2016. Free opercula were collected from the forest floor after shedding, in a radius of 1.5 m around each tree trunk, while capsules were harvested from tree crowns. Opercula were sampled from 89 and 14 trees (103 total) and capsules were sampled from 87 and 136 trees (223 total) from the Boyer and Geeveston trials, respectively. Collected capsules were air dried before measurement to ease the opening of valves and removal of seeds and chaff. Ten opercula and five capsules per tree were measured with callipers and averaged for analysis. Differences and ratios derived from these measurements were calculated based on tree averages.

**Fig. 1.**
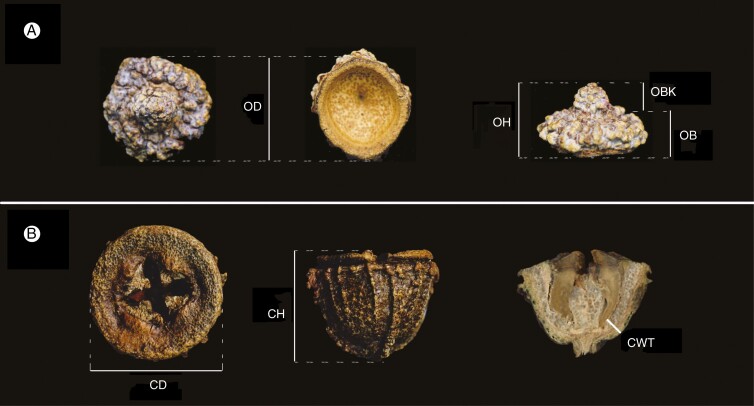
Traits measured on reproductive structures of *Eucalyptus globulus*. (A) Measurements on operculum: diameter (OD), height (OH), base height (OB) and beak height (OBK). (B) Measurements on capsule: diameter (CD), height (CH) and wall thickness (CWT) measured on a cut capsule.

Several aspects of operculum morphology were examined (Table 1, Fig. 1A). Operculum diameter (OD) was measured across the widest point of the base of the operculum, while operculum height (OH) was measured from the bottom of the operculum to the top of the operculum beak. The distance from the bottom of the operculum to the beginning of the beak was also measured and termed operculum base height (OB). Beak height (OBK) was calculated as the difference between the operculum height and the operculum base height. Two measurements of operculum shape were used in this study. The first was a ratio between operculum height and diameter, used to quantify the whole shape of the operculum (OS). The second followed Kirkpatrick’s (1975) classification of the operculum beak, from which was calculated the proportion of opercula per tree that did not exhibit flat beaks (i.e. classes 1, 3 and 4 in Kirkpatrick’s classification), which we called opercula shape by class (OSC).

**Table 1.** The operculum and capsule traits measured in an F_2_ family of *Eucalyptus globulus*, number of trees sampled and statistics based on the tree-level averages.

Trait	Code	Description	Unit	*n*	Mean	SD	Min.	Max.
Operculum diameter	OD	Length of the widest point of the base of the operculum	mm	103	16.2	1.37	13.6	19.4
Operculum height	OH	Length from the bottom to the top of the operculum	mm	103	10.1	1.2	7.3	12.9
Operculum shape	OS	Ratio between operculum height and diameter		103	0.6	0.07	0.4	0.8
Operculum shape by class	OSC	Proportion of opercula per tree that did not exhibit flat beaks	%	103	59.4	36.91	0	100
Operculum base height	OB	Length from the base to the beginning of the beak in the operculum	mm	103	5.5	0.59	4.0	7.2
Operculum beak height	OBK	Length of the operculum beak	mm	103	4.5	0.84	2.3	6.9
Capsule diameter	CD	Length of the widest point of the capsule disc	mm	206	20.7	1.57	16.5	24.5
Capsule height	CH	Length from the end of the pedicel to the capsule disc	mm	204	13.9	1.21	10.7	16.9
Capsule shape	CS	Ratio between capsule height and diameter		204	0.7	0.06	0.5	0.9
Capsule weight	CW	Averaged mass of an individual capsule	g	223	2.4	0.46	1.2	3.7
Capsule wall thickness	CWT	Length of the narrowest point of the capsule wall	mm	204	3.0	0.41	2.1	4.3

Likewise, several measures of capsule morphology were taken (Table 1, Fig. 1B). Capsule diameter (CD) was measured across the widest point of the disc, while capsule height (CH) was measured from the disc to the bottom of the capsule excluding the length of the pedicel. Capsule weight (CW) was measured after seed extraction as the weight of total capsule collection divided by the number of capsules collected per tree. Capsule wall thickness (CWT) was measured at the narrowest point of one half of each capsule obtained after cutting them through the centre with a bandsaw. The ratio of capsule height to diameter was also used to give a measure of the overall shape of capsules (CS). Pearson’s correlations were calculated between all traits based on the tree averages to explore phenotypic associations among traits.

### QTL analysis

To examine the genetic architecture of operculum and capsule traits, QTL analysis was performed on the F_2_ family with the software MapQTL 6 (Van Ooijen, 2009), using the tree averages of operculum and capsule traits and the linkage map developed by Hudson *et al.* (2012). To decrease computational demand, the linkage map was reduced to 480 markers located at intervals of 2–5 cM by removing most 3 : 1 segregating Diversity Arrays Technology (DArT) markers and retaining all simple sequence repeat (SSR) markers and an even distribution of DArT markers segregating in a 1 : 1 ratio. Permutation tests (1000 permutations) were run to determine the significance thresholds of the logarithm of odds (LOD) at the genome-wide and chromosome-wide levels (Churchill and Doerge, 1994). Putative QTL were declared as significant (i.e. genome-wide type I error rate <0.05) or suggestive (i.e. chromosome-wide type I error rate <0.05) depending on LOD score (Freeman *et al.*, 2008). Initially, interval mapping was applied using site as an experimental design cofactor. When QTL peaks exceeded the suggested LOD threshold in interval mapping, the closest markers were added as cofactors in a subsequent analysis by restricted multiple-QTL model (rMQM) mapping. The rMQM analyses were conducted using an iterative approach until no more QTL were detected, cofactor markers were the closest marker to each QTL, and QTL positions were stable (Van Ooijen, 2009).

### Identification of putative candidate genes

To identify candidate genes, every QTL peak marker associated with operculum and capsule traits was positioned in the *E. grandis* reference genome via a BLAST search of the associated marker sequences in Phytozome 13 (https://phytozome-next.jgi.doe.gov/blast-search). This was possible because almost all markers had DNA sequences associated with them, as either the primer sequences for microsatellites or the sequenced DArT markers (Sansaloni *et al.*, 2011). Following that, an extensive search of genes implicated in petal and fruit development of the model plant *Arabidopsis thaliana* was done from the literature (see Supplementary Data Table S1). The sequence of each of these 94 genes was BLAST searched against the *E. grandis* reference genome to identify eucalypt orthologues (by accepting hits to annotated genes at a threshold of e-value <1e^−3^). The putative functions of these genes were taken from Phytozome 13 or the original references. The eucalypt orthologues of the arabidopsis genes which were within 2.5 Mbp of the QTL peak marker position were accepted as putative candidate genes.

### Assessment and analysis of flower buds

**Fig. 2.**
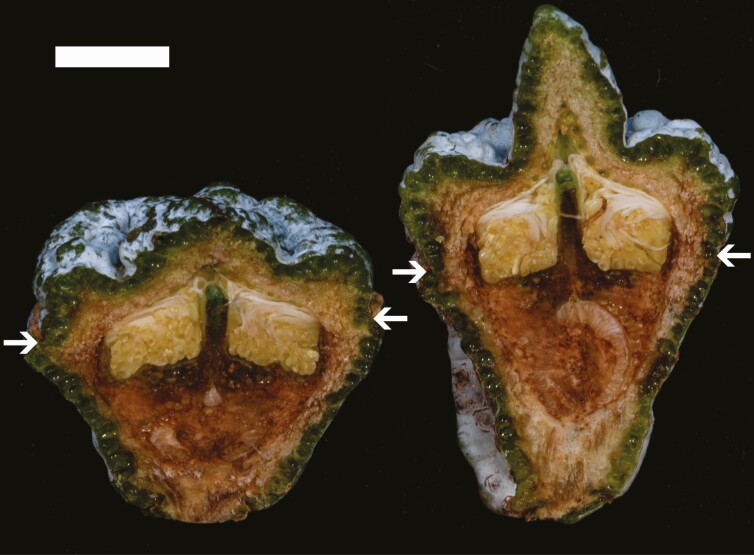
Longitudinal sections of phenotypically distinctive flower buds of *Eucalyptus globulus*. On the left, bud with flat operculum beak. On the right, bud with tall operculum beak. Note the coordinated texture, waxiness and colour of the operculum and the receptacle. Scale bar = 5 mm. Arrows show the suture point where the operculum (above the arrows) joins the receptacle.

Flower bud measurements were undertaken to examine the hypotheses that (1) operculum beak size was associated with style length and (2) the association between operculum and the developing capsule starts early in flower bud development. Flower buds were collected from two groups of phenotypically distinctive F_2_ trees growing at Boyer during autumn 2021 (*n* = 7). One group had four trees with distinctive operculum beaks, while the other group comprised three trees with small beaks and opercula that were effectively flat (Fig. 2). Four to eight buds per tree were cut longitudinally, one half of each bud was scanned and style length was measured with the software ImageJ v.1.52a (Schneider *et al.*, 2012). Operculum shape and receptacle shape (equivalent to capsule shape) were measured on flower buds as described above for OS and CS. To determine the statistical significance of the observed differences between the two group of trees, a mixed model was fitted to the individual bud data with group fitted as a fixed effect and tree nested within group fitted as a random effect. Analyses were performed with the R statistical language v.3.6.3 (R Core Team, 2020) using the package lme4 (Bates *et al.*, 2015) and the significance of the fixed group effect was determined using a likelihood ratio test (LRT). Additionally, Pearson’s correlations were calculated between flower bud traits based on tree averages using the R-package Hmisc (Harrell and Dupont, 2021).

## RESULTS

**Fig. 3.**
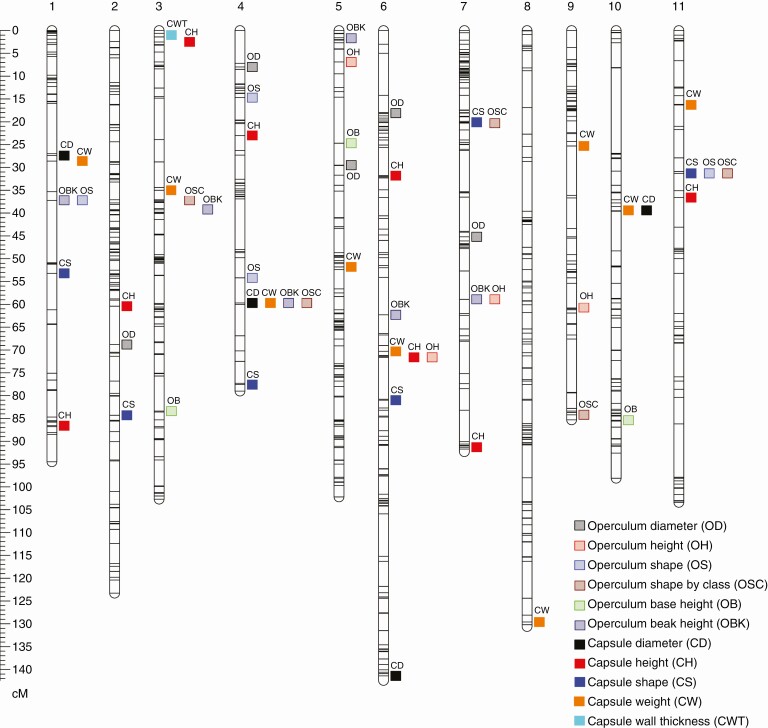
Quantitative trait loci (QTL) associated with operculum and capsule traits in *Eucalyptus globulus*. Marker positions on the linkage map are shown as lines on the 11 linkage groups. Linkage group number and orientation is as per the *E. grandis* reference genome (Myburg *et al.*, 2014).

QTL analysis allowed the detection of 55 QTL across all operculum and capsule traits, of which 28 were significant and 27 were suggestive (Tables 2 and 3). The number of QTL detected per trait ranged from one to nine, and the percentage of variance explained (PVE) by each QTL ranged from 2.9 to 26.4 %. Overall, 39 regions of the genome (here defined as LOD peaks more than 5 cM apart) were detected across the 11 linkage groups of *E. globulus* (Fig. 3).

**Table 2.** Putative quantitative trait loci (QTL) for operculum traits in an F_2_ population of *Eucalyptus globulus*

Trait	ID^a^	LG^b^	cM^c^	Marker^d^	LOD^e^	PVE^f^
Operculum diameter (OD)	OD7	7	45.2	643269	5.58**	14.7
	OD5	5	28.9	573064	5.37**	13.9
	OD2	2	68.8	641024	4.46*	11.3
	OD6	6	18.2	568290	3.47	7.4
	OD4	4	8.0	640171	3.29	8.1
Operculum height (OH)	OH6	6	71.6	563633	6.46***	18.1
	OH5	5	6.2	565220	4.37*	11.7
	OH7	7	58.9	637768	3.73	9.8
	OH9	9	60.8	641541	3.50	9.2
Operculum shape (OS)	OS11	11	31.3	566749	9.35***	26.4
	OS1	1	37.2	574251	4.76**	12.0
	OS4a	4	14.7	638422	4.39*	10.5
	OS4b	4	54.2	564417	3.51	8.6
Operculum shape by class (OSC)	OSC11	11	31.3	566749	5.88***	13.2
	OSC4	4	59.7	570676	5.81***	12.9
	OSC9	9	84.2	Embra204	3.54	7.5
	OSC7	7	20.3	564872	3.30	7.0
	OSC3	3	37.2	641763	3.41	7.2
Operculum base height (OB)	OB5	5	24.0	574595	5.31**	13.5
	OB10	10	85.4	571236	4.37*	9.7
	OB3	3	83.4	600338	3.46	7.5
Operculum beak height (OBK)	OBK3	3	39.2	570900	7.01***	13.5
	OBK6	6	62.3	571092	5.89**	11.4
	OBK5	5	0	572752	5.26**	9.9
	OBK4	4	59.7	570676	4.37*	8.1
	OBK1	1	37.2	574251	4.23	7.9
	OBK7	7	58.9	637768	3.93	6.9

^a^Name of the QTL,

^b^linkage group,

^c^position of QTL on a linkage group in centimorgans,

^d^marker closest to the QTL peak,

^e^significance,

^f^percentage of variance explained. Genome-wide significance is indicated by asterisks: ***P < 0.001, **P < 0.01, *P < 0.05. The remaining QTL were classified as suggestive (chromosome-wide type I error rate <0.05).

**Table 3.** Putative quantitative trait loci (QTL) for capsule traits in an F_2_ population of *Eucalyptus globulus*

Trait	ID^a^	LG^b^	cM^c^	Marker^d^	LOD^e^	PVE^f^
Capsule diameter (CD)	CD6	6	141.4	641567	5.42**	9.0
	CD4	4	59.7	570676	4.42*	7.3
	CD10	10	39.5	Embra153	3.55	5.8
	CD1	1	27.4	599787	3.52	5.7
Capsule height (CH)	CH11	11	36.6	Eg99	9.01***	11.3
	CH4	4	23.0	571676	8.09***	10.0
	CH7	7	91.3	503736	6.27**	7.6
	CH2	2	60.4	503579	4.37*	5.2
	CH3	3	2.5	573728	4.15	4.9
	CH6a	6	71.6	563633	3.98	4.9
	CH6b	6	31.8	639232	3.77	4.7
	CH1	1	86.6	644056	3.67	4.3
Capsule shape (CS)	CS11	11	31.3	566749	11.80***	16.4
	CS1	1	53.2	Embra222	5.38**	7.1
	CS2	2	84.3	Embra27	4.66*	6.1
	CS7	7	20.1	565241	4.27*	5.6
	CS6	6	81.0	565210	4.54*	5.9
	CS4	4	77.6	565463	2.99	3.8
Capsule weight (CW)	CW5	5	51.1	644217	9.04***	10.4
	CW10	10	39.5	Embra153	7.94***	9.0
	CW4	4	59.7	570676	6.95***	7.8
	CW9	9	25.3	505052	5.66**	6.3
	CW6	6	70.3	Embra173	4.50*	4.9
	CW8	8	129.6	Es76	4.12	4.5
	CW3	3	35.0	640311	3.92	4.3
	CW1	1	28.6	Eg65	3.69	4.0
	CW11	11	16.3	571646	2.75	2.9
Capsule wall thickness (CWT)	CWT3	3	0	642915	4.07	7.7

^a^Name of the QTL,

^b^linkage group,

^c^position of QTL on a linkage group in centimorgans,

^d^marker closest to the QTL peak,

^e^significance,

^f^percentage of variance explained. Genome-wide significance is indicated by asterisks: ***P < 0.001, **P < 0.01, *P < 0.05. The remaining QTL were classified as suggestive (chromosome-wide type I error rate <0.05).

In total, 27 QTL were detected for operculum traits, of which 16 were significant and 11 suggestive (Table 2). These QTL mapped to 21 regions of the genome in ten different linkage groups (Fig. 3). The number of QTL that influenced each operculum trait ranged from three to six, explaining 30.7–57.7 % of the total variance of these traits. Likewise, 28 QTL were detected for variation in capsule traits, of which 16 QTL were declared significant and 12 suggestive (Table 3). These QTL occupied 20 genomic regions on 11 linkage groups and when combined within trait explained 7.7–54.1 % of the total variance of capsule traits. Several co-locating QTL for capsule and operculum traits were observed, which is not unexpected given the high correlation between specific traits. For example, an operculum beak height (OBK) QTL co-located with an operculum height (OH) QTL at 58.9 cM in linkage group 7, reflecting the strong phenotypic correlation of these traits (Pearson’s *r* = 0.89, Table 4).

**Table 4.** Pearson correlations among operculum and capsule traits for the F_2_ population of *Eucalyptus globulus*

	OD	OH	OS	OSC	OB	OBK	CD	CH	CS	CW
OH	**0.46** ^***^									
OS	−0.27 ^**^	**0.72** ^***^								
OSC	−0.19 n.s.	**0.52** ^***^	**0.69** ^***^							
OB	**0.62** ^***^	**0.77** ^***^	**0.38** ^***^	0.21 *						
OBK	0.22 *	**0.89** ^***^	**0.79** ^***^	**0.60** ^***^	**0.40** ^***^					
CD	0.30 *	0.05 n.s.	−0.12 n.s.	−0.14 n.s.	0.12 n.s.	−0.01 n.s.				
CH	0.06 n.s.	0.32 *	0.29 *	0.14 n.s.	0.22 n.s.	0.31 *	**0.41** ^***^			
CS	−0.20 n.s.	0.28 n.s.	0.42 ^**^	0.26 n.s.	0.11 n.s.	0.33 *	−0.44 ^***^	**0.63** ^***^		
CW	**0.58** ^***^	0.22 *	−0.16 n.s.	−0.17 n.s.	**0.40** ^***^	0.04 n.s.	**0.49** ^***^	**0.41** ^***^	−0.04 n.s.	
CWT	0.30 *	0.15 n.s.	−0.02 n.s.	−0.06 n.s.	0.05 n.s.	0.18 n.s.	**0.36** ^***^	0.24 ^***^	−0.06 n.s.	**0.40** ^***^

Operculum traits included diameter (OD), height (OD), shape (OS and OSC), base height (OB) and beak height (OBK). Capsule traits included diameter (CD), height (CH), shape (CS), weight (CW) and wall thickness (CWT). Correlations were calculated using tree-level averages. Statistical significance is indicated by asterisks:

^***^ *P* < 0.001,

^**^ *P* < 0.01,

*P < 0.05, n.s. not significant. Numbers in bold indicate statistical significance after Bonferroni correction for multiple comparisons (*P* < 0.05/50).

**Fig. 4.**
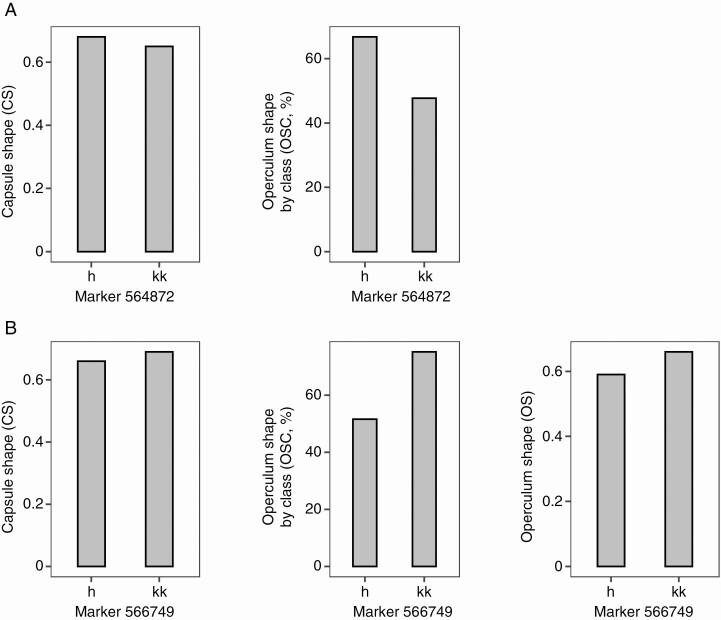
Genotype average (h and kk) from the closest marker to the co-located QTL on linkage group 7 (A) and 11 (B) for capsule shape (CS) and operculum shape (both OS and OSC).

Co-location of QTL affecting both capsule and operculum morphology was found in several regions of the *E. globulus* genome. Of particular note was the co-location of capsule shape (CS) and operculum shape (OS and OSC) QTL at 20.3 cM in linkage group 7 and 31.3 cM in linkage group 11. Analysis of the genotype means at the marker closest to these QTL revealed a common direction of variation, resulting in a more elongated (tall and narrow) or more compact (short and wide) form in both operculum and capsule (Fig. 4). Other co-locations between capsule structure (i.e. CD, CH and CW) and operculum dimensions and shape (i.e. OH, OBK, OS and OSC) also suggest the same direction of variation for operculum and capsule morphology (Supplementary Data Fig. S1).

Co-locations between QTL and eucalypt orthologues of arabidopsis flower and fruit genes revealed 26 candidate genes for 17 QTL regions (Table 5). Annotated functions for these genes included transcription factors, and regulators of organ shape, plant metabolism, and cell proliferation and expansion. Genes with annotated functions of cell proliferation and expansion were the most common (results not shown). The number of candidate genes per QTL varied from one to five for operculum traits and from one to three for capsule traits. Two arabidopsis genes (*YAB2* and *CYP78A9*) hit genome positions near QTL of both operculum and capsule traits, including operculum and capsule shape.

**Table 5.** Co-location of operculum and capsule trait QTL in *Eucalyptus globulus* with *Eucalyptus grandis* orthologues of petal and fruit development genes in *Arabidopsis thaliana*

Trait	QTL^a^	Arabidopsis gene ID	Arabidopsis alias(es)	*Eucalyptus grandis* gene ID	Position (bp)^b^	Distance (bp)^c^
Operculum diameter (OD)	OD2	AT5G28640	*ANGUSTIFOLIA3, AN3*	Eucgr.B02127.1	40 275 241	2 020 238
	OD2	AT2G28610	*WUSCHEL*, *WUS*	Eucgr.B02435.1	43 981 689	1 686 210
	OD4	AT5G37020	*ARF8*	Eucgr.D00264.1	4 456 430	2 296 386
Operculum height (OH)	OH5	AT5G06070	*RABBIT EARS*, *RBE*	Eucgr.E00283.1	2 702 713	918 047
	OH7	AT1G24120	*ARGOS-LIKE 1*, *ARL1*	Eucgr.G02304.1	43 681 977	1 754 646
	OH7	AT1G13400	*NUBBIN*, *NUB*	Eucgr.G02316.1	43 823 087	1 613 536
	OH7	AT1G68640	*PERIANTHIA*, *PAN*	Eucgr.G02341.1	44 080 267	1 356 356
	OH7	AT5G60970	*TCP5*	Eucgr.G02354.1	44 219 050	1 217 573
Operculum shape by class (OSC)	OSC3 (OBK3, CW3)	AT1G08465	*YAB2*	Eucgr.C01515.1	21 167 250	1 647 256
	OSC9	AT4G13890	*SHM5*	Eucgr.I02741.1	38 303 872	41 926
	OSC9	AT1G22020	*SHM6*	Eucgr.I02741.1	38 303 202	41 256
Operculum beak height (OBK)	OBK4 (OS, OSC, CD, CW)	AT4G17810	*SUPERMAN*, *SUP*	Eucgr.D02189.1	35 501 006	788 165
	OBK5	AT3G61880	*CYP78A9*	Eucgr.E00023.1	283 251	628 925
Capsule diameter (CD)	CD1 (CW1)	AT4G36920	*APETALA2*, AP2	Eucgr.A01182.1	24 434 809	231 793
	CD10 (CW10)	AT5G03680	*PETAL LOSS*, *PTL*	Eucgr.J01012.1	10 959 765	1 566 797
	CD10 (CW10)	AT3G63530	*BIG BROTHER*, *BB, BB2*	Eucgr.J01268.1	13 817 448	1 290 943
Capsule height (CH)	CH3	AT2G26330	*ERECTA*, *ER*	Eucgr.C00732.1	12 647 336	996 656
	CH3	AT5G62230	*ERL1*	Eucgr.C00732.1	12 647 417	995 946
	CH6b	AT3G15030	*TCP4*	Eucgr.F01204.1	16 222 224	1 036 547
	CH6b	AT2G31070	*TCP10*	Eucgr.F01204.1	16 222 688	1 036 083
Capsule shape (CS)	CS2	AT2G33860	*ETTIN*, *ETT*	Eucgr.B02480.1	44 872 757	974 675
	CS2	AT5G60690	*REVOLUTA*, *REV*	Eucgr.B02504.1	45 073 481	773 951
	CS4	AT1G08465	*YAB2*	Eucgr.D02474.1	38 561 207	901 330
	CS4	AT2G45190	*AFO*, *FIL*	Eucgr.D02474.1	38 561 394	901 142
	CS4	AT3G61880	*CYP78A9*	Eucgr.D02644.1	40 325 072	861 769
	CS6	AT1G13710	*KLUH*, *CYP78A5*	Eucgr.F02654.1	38 740 558	683 679
	CS6	AT1G43850	*SEUSS*	Eucgr.F02753.1	39 633 179	207 307
Capsule weight (CW)	CW11	AT2G20825	*ULT2*	Eucgr.K00939.1	11 642 380	554 015
	CW11	AT4G28190	*ULT1*	Eucgr.K00939.1	11 642 488	553 907
Capsule wall thickness (CWT)	CWT3	AT4G37840	*HKL3*	Eucgr.C00559.1	8 851 889	2 235 736

^a^Parentheses indicate co-location with another QTL or a close QTL in the F_2_ population.

^b^Midpoint of the *Eucalyptus grandis* orthologue in the *E. grandis* reference genome.

^c^Distance between gene midpoint and the midpoint of the QTL peak marker sequence in the *Eucalyptus grandis* reference genome.

Sampling of flower buds from trees with extreme beaked or flat opercula within the F_2_ populations at Boyer was undertaken to test whether operculum shape was related to style length and to receptacle shape. The two groups sampled were highly significantly different in operculum shape (LRT, *G *= 10.37, d.f. = 1, *P* < 0.001), but did not differ significantly in style length (LRT, *G *= 0.14, d.f. = 1, *P* = 0.70). While the difference in shape of the receptacle between groups was not statistically significant (LRT, *G *= 2.49, d.f. = 1, *P* = 0.11), operculum shape was moderately correlated with the shape of the receptacle (*r* = 0.69, *P* = 0.08) in flower buds. Considered together with the similar positive correlation for capsule shape and mature operculum shape (Table 4), this suggests a link between the traits that starts in early bud development.

## DISCUSSION

Most QTL studies in *Eucalyptus* to date have focused on traits of economic importance such as growth and wood quality (Thumma *et al.*, 2010; Gion *et al.*, 2011; Freeman *et al.*, 2013; Resende *et al.*, 2016) and resistance to pests and disease (Alves *et al.*, 2012; Zarpelon *et al.*, 2015; Rosado *et al.*, 2016; Mhoswa *et al.*, 2020). However, a few studies have addressed other traits related to physiological and/or ecological functions, such as foliar terpene and cuticular wax composition, heterochrony and drought resistance (Freeman *et al.*, 2008; Hudson *et al.*, 2014; Gosney *et al.*, 2016; Ammitzboll *et al.*, 2020). The present study is the first to examine the genetic architecture underlying flower and fruit morphology in the genus. We show that operculum and capsule shape and size are under genetic control in *E. globulus*, and identify a significant number of QTL across all traits, many of which are co-located. Within organs, most of the co-located QTL probably represent allometric or simple dependent relationships among traits (e.g. capsule weight and diameter). However, QTL co-locations between operculum and capsule traits were more interesting and suggest that elements of operculum and capsule morphology share a common genetic pathway. This association might be expressed early during bud development, given the observations made on flower buds. We identify several candidate genes near QTL that may underlie the variation in operculum and capsule morphology in *E. globulus*, some of which may affect both organs. These candidate genes may be the key to understanding the genetic control of the taxonomically and ecologically important variation in these organs in eucalypts. Of interest are flower development genes under operculum QTL that may have acquired novel functions given the operculum is a highly derived petaline structure. There is also the challenge of identifying the genes underlying QTL where no arabidopsis flowering genes were found, particularly the major QTL co-location on linkage group 11.

The genetic control of operculum and capsule morphology may best be considered as polygenic, given that QTL were distributed over all the 11 linkage groups of *E. globulus*. While the possibility of an inflated QTL effect (PVE) with the population size studied cannot be dismissed (Beavis, 1998), these traits are probably under strong genetic control given the total combined PVE for some traits exceeded 50 %. There were multiple co-locations between QTL underlying different traits within each organ, for example operculum height, beak and shape, and QTL underlying capsule weight, diameter, height and wall thickness. When taken with the strong positive correlations between many of these traits, these QTL probably represent pleiotropic growth loci affecting the general allometry of these organs. For example, a prominent operculum beak was often associated with a higher and more elongated operculum, which is supported by the co-location between QTL for operculum height and beak height on linkage group 7. While other QTL have been discovered in this specific cross of *E. globulus*, variously associated with growth, flowering time, heterochrony, response to pathogens, and resistance to and recovery from drought (Hudson *et al.*, 2014; Butler *et al.*, 2016; Ammitzboll *et al.*, 2018, 2020), the reproductive morphology QTL we describe here are among the strongest detected. Outside of *Eucalyptus*, several studies document QTL for size and shape of nuts, drupes or hesperidia in other tree species (Fernández i Martí *et al.*, 2013; da Silva Linge *et al*., 2015; Yu *et al.*, 2016; Frary *et al.*, 2019), but to our knowledge no other research has focused on the genetic architecture of fruit size and shape in forest trees.

A number of promising candidate genes were associated with QTL for operculum morphology. QTL associated with the operculum were often near *Eucalyptus* orthologues of arabidopsis genes linked to the control of petal growth. This makes sense as the inner operculum of *Eucalyptus* is a sympetalous corolla (Drinnan and Ladiges, 1988). For example, QTL associated with operculum height are near the eucalypt orthologues for *NUBBIN*, *ARL1* and *TCP5*, which are associated with mechanisms of cell proliferation or cell expansion in petals of arabidopsis (Dinneny *et al.*, 2006; Delgado-Benarroch *et al.*, 2009; Chen *et al.*, 2018; van Es *et al.*, 2018). Similarly, development of the operculum as a whole and its beak specifically might be triggered by members of the C2H2 zinc finger family *RABBIT EARS* and *SUPERMAN*, which encode cell division during early stages of petal growth in *A. thaliana* (Huang and Irish, 2015). In addition, QTL for operculum diameter are near to orthologues of *ARF8* and *WUSCHEL*, which are known to regulate auxin signalling, with the former affecting late phases of petal growth (Huang and Irish, 2015; Ma *et al.*, 2019). Interestingly, an orthologue of *ANGUSTIFOLIA3*, which is known to affect the width of flowers in mutants of *A. thaliana* (Anastasiou *et al.*, 2007), is near a QTL for operculum diameter, suggesting a similar role in *E. globulus* flower width. These genes are therefore promising candidates for control of operculum development and morphology.

Surprisingly, only two fruit development-specific orthologues from arabidopsis co-located with capsule trait QTL. These included an orthologue for *APETALA2*, associated with establishment of the floral meristem and fruit growth (Ripoll *et al.*, 2011), and an orthologue of *HKL3*, which is known to control development and maturation of fruits (Zhang *et al.*, 2017). Given that the arabidopsis fruit is a silique but the eucalypt fruit is a woody capsule formed by the growth of the fertilized flower and the emergence and lignification of fibres, filiform sclereids and brachysclereids in the receptacle (Bohte and Drinnan, 2011), it is quite likely that a different suite of genes would be required during its development, which might be reflected in a relatively low representation of known arabidopsis fruit development genes in *Eucalyptus* QTL regions. However, many arabidopsis petal and flower development orthologues also co-located with capsule trait QTL, including *PETAL LOSS*, *BIG BROTHER* and *ERL1* (Chandler *et al.*, 2011; Lampugnani *et al.*, 2012; Abraham *et al.*, 2013). While it is possible that these genes are merely genetically associated with fruit/capsule characteristics in *Eucalyptus*, it is also possible that at least in some cases they have been co-opted into capsule development pathways, given their functional links to control of cell proliferation. For instance, several capsule size QTL co-locate with orthologues of genes such as *ERECTA*, *ULT1*, *ULT2*, *TCP4* and *TCP10*, which have not only been associated with petal development but also with cell proliferation of other arabidopsis organs (Shpak *et al.*, 2004; Nag *et al.*, 2009; Monfared *et al.*, 2013), although not specifically fruit. There are also several cases where capsule and operculum QTL include different candidate genes that are members of the same family. For example, the arabidopsis YABBY genes affect the abaxial cell fate of flowering meristems (Bowman, 2000) and eucalypt orthologues of YABBY gene family members (*YAB2* and *FIL*) were identified near QTL of capsule and operculum shape. Similarly, orthologues of genes in the cytochrome P450 family, *CYP78A5* and *CYP78A9*, are located close to QTL for capsule shape and other operculum traits. This gene family has been shown to affect cell proliferation in arabidopsis petals and other organs during later stages of development (Abraham *et al.*, 2013). This suggests that elements of operculum and capsule development could be influenced by the same genes, or similar genetic pathways.

The co-location of QTL associated with operculum and capsule traits provides broad support for the hypothesis of shared genetic control of elements of capsule and operculum morphology. Notably, the two strongest QTL were associated with capsule shape and operculum shape, and were co-located on linkage group 11. The common directionality of alleles for these QTL suggests that this relationship is probably one of pleiotropy, with a single gene influencing a generalized elongated or compact floral structure, presumably expressed early in development. However, no eucalypt orthologues to the petal and fruit development genes of arabidopsis were found close to the two QTL co-locations affecting capsule and operculum shape on linkage groups 7 and 11. A common developmental link between the operculum and the bud receptacle, which eventually becomes the woody capsule, has been previously suggested. Drinnan and Ladiges (1989) found that lateral dimensions of the operculum appear to approach those of the bud width while the bud is still quite small in the Eudesmieae B lineage of eucalypts and *E. caesia* and suggested that interactions between the growth rate of the operculum and receptacle contributed to the eventual size of the developing flower. Coordination of texture, waxiness and colour for operculum and receptacle is a feature of *E. globulus* (see Fig. 2) and this supports the idea of common developmental control for both structures, although this relationship might not be constant for all the species of the genus (e.g. *E. erythrocorys* has a bright red operculum but a green receptacle). These aspects of reproductive structure development are difficult to investigate thoroughly, as correlations between most quantitative operculum and capsule traits are low to moderate, probably due to stress or fertilization success affecting capsule development.

Overall, this QTL study shows that variation in both operculum and capsule shape and size are under genetic control in *E. globulus*. It is likely that there is a common genetic pathway controlling the development of both the operculum and capsule, involving both pleiotropy and utilization of common gene families.

## SUPPLEMENTARY INFORMATION

Supplementary data are available at *Annals of Botany* online and consist of the following. Table S1: list of genes associated with floral and fruit development in *Arabidopsis thaliana* blasted in the *Eucalyptus grandis* reference genome. Fig. S1: genotype average of co-located operculum and capsule traits for different alleles.

mcac072_suppl_Supplementary_Figure

mcac072_suppl_Supplementary_Table
