# Supplementary material for: Genetic control of the operculum and capsule morphology of Eucalyptus globulus
Source: Ann Bot. 2022 Jun 2;130(1):97–108. doi: 10.1093/aob/mcac072 (PMC9295918; doi:10.1093/aob/mcac072)
Supplement: mcac072_suppl_Supplementary_Figure [file mcac072_suppl_supplementary_figure.doc]

**
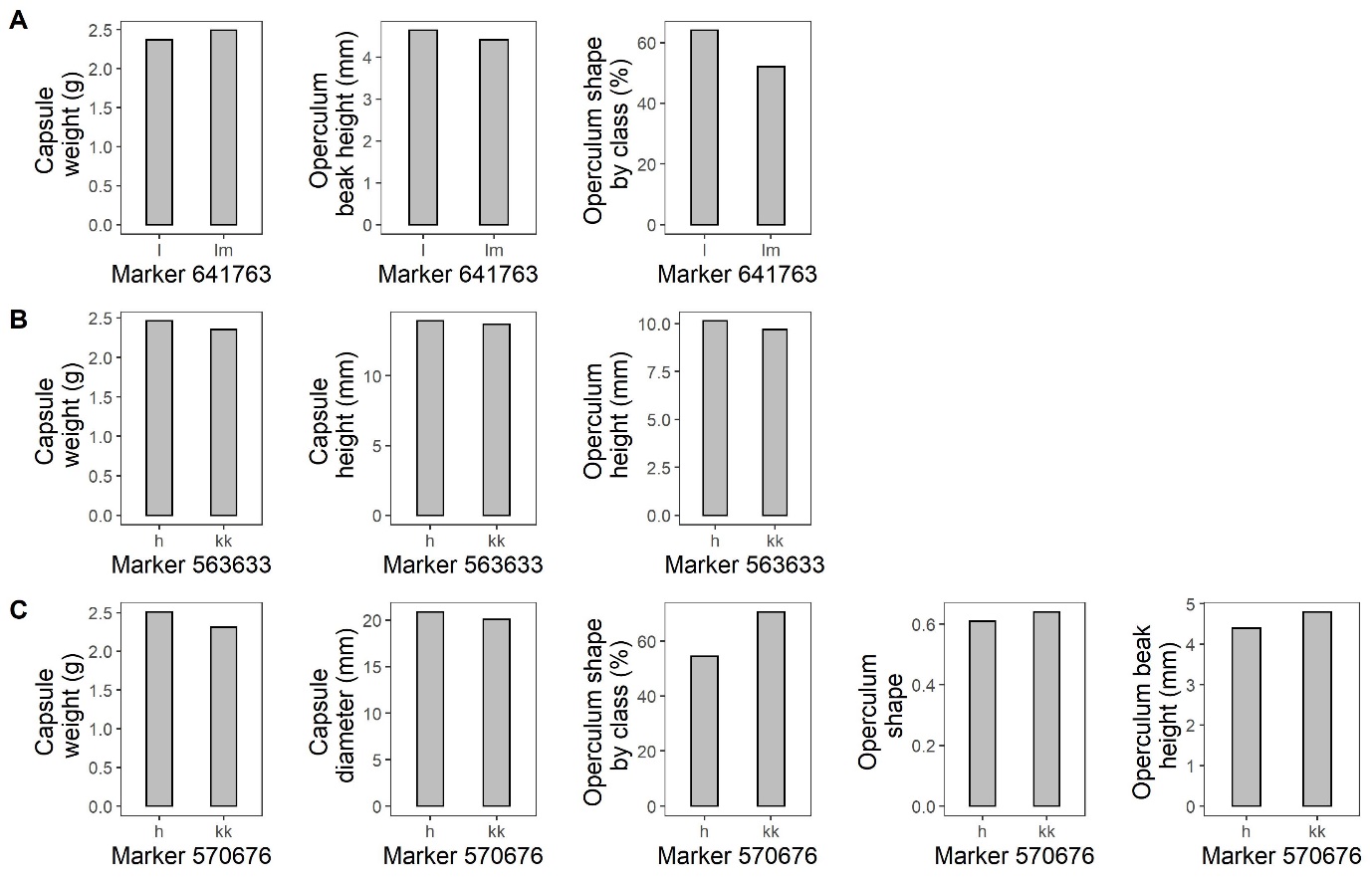
**

**Figure S1.** Genotype average of co-located operculum and capsule traits for different alleles. A. Co-locations for traits capsule weight, operculum beak height and operculum shape by class in linkage group 3. B. Co-locations for traits capsule weight, capsule height and operculum height in linkage group 6. C. Co-locations for traits capsule weight, capsule diameter, operculum shape by class, operculum shape and operculum beak height in linkage group 4.
