# Supplementary material for: Genetic control of the operculum and capsule morphology of Eucalyptus globulus
Source: Ann Bot. 2022 Jun 2;130(1):97–108. doi: 10.1093/aob/mcac072 (PMC9295918; doi:10.1093/aob/mcac072)
Supplement: mcac072_suppl_Supplementary_Table [file mcac072_suppl_supplementary_table.doc]

**Table S1.** List of genes associated with floral and fruit development in *Arabidopsis thaliana* (references in footnote) blasted in the *Eucalyptus grandis* reference genome

| **Arabidopsis Gene ID** | **Alias(es)** | **Position in the arabidopsis genome (TAIR10, bp)** |
| --- | --- | --- |
| AT2G45190 | *ABNORMAL FLORAL ORGANS, FIL* | Chr2:18628264..18630712 reverse |
| AT4G18960 | *AGAMOUS, AG* | Chr4:10382855..10388539 forward |
| AT5G10510 | *AINTEGUMENTA, ANT* | Chr5:3315529..3320202 forward |
| AT5G10510 | *AINTEGUMENTA-LIKE 6, AIL6* | Chr5:3315529..3320202 forward |
| AT5G67110 | *ALCATRAZ, ALC* | Chr5:26785106..26786383 reverse |
| AT5G28640 | *ANGUSTIFOLIA3, AN3* | Chr5:10647570..10649892 reverse |
| AT4G36920 | *APETALA 2, AP2* | Chr4:17400847..17403332 forward |
| AT3G50860 | *APETALA 3, AP3* | Chr3:18901997..18904197 forward |
| AT5G62000 | *ARF2* | Chr5:24910358..24915210 forward |
| AT1G72350 | *ARF6* | Chr1:27239272..27239947 reverse |
| AT5G37020 | *ARF8, AUXIN RESPONSE FACTOR8* | Chr5:14630027..14634387 forward |
| AT3G59900 | *ARGOS* | Chr3:22129726..22130464 forward |
| AT1G24120 | *ARGOS-LIKE 1, ARL1* | Chr1:8529143..8532212 reverse |
| AT1G65620 | *AS2* | Chr1:24398925..24400969 forward |
| AT2G22660 | *At2g22660* | Chr2:9627407..9631081 forward |
| AT5G63310 | *ATNDPK2* | Chr5:25371903..25373861 reverse |
| AT1G75950 | *ATSKP1* | Chr1:28516547..28517699 forward |
| AT5G02030 | *BELLRINGER, BLR* | Chr5:395633..399041 forward |
| AT3G63530 | *BIG BROTHER, BB, BB2* | Chr3:23456157..23458459 reverse |
| AT3G57130 | *BOP1* | Chr3:21147835..21150027 forward |
| AT1G59640 | *BPEp, BIG PETAL P* | Chr1:21909180..21911201 reverse |
| AT5G21482 | *CKX7* | Chr5:7226726..7230210 forward |
| AT1G75820 | *CLAVATA1, CLV1* | Chr1:28463462..28466971 reverse |
| AT2G27250 | *CLAVATA3, CLV3* | Chr2:11664985..11665769 reverse |
| AT1G04400 | *CRY2* | Chr1:1185549..1188517 reverse |
| AT3G60100 | *CSY5* | Chr3:22192729..22196447 forward |
| AT3G15170 | *CUC1* | Chr3:5109883..5111447 forward |
| AT5G53950 | *CUC2* | Chr5:21901966..21903823 reverse |
| AT1G76420 | *CUC3* | Chr1:28672029..28673934 reverse |
| AT4G34160 | *CYCD3* | Chr4:16357639..16359555 forward |
| AT3G61880 | *CYP78A9* | Chr3:22905868..22907958 reverse |
| AT3G61880 | *CYP78A9* | Chr3:22905868..22907958 reverse |
| AT1G19270 | *DA1* | Chr1:6662501..6666028 forward |
| AT1G24590 | *DORNRÖSCHEN- LIKE, DRNL* | Chr1:8714389..8715309 reverse |
| AT2G26330 | *ERECTA, ER* | Chr2:11208183..11213971 reverse |
| AT5G62230 | *ERL1* | Chr5:24996234..25002340 forward |
| AT5G07180 | *ERL2* | Chr5:2227571..2233417 reverse |
| AT2G33860 | *ETTIN, ETT* | Chr2:14325269..14328978 reverse |
| AT3G25110 | *FATA1* | Chr3:9146270..9148486 reverse |
| AT4G13050 | *FATA2* | Chr4:7617560..7619616 forward |
| AT5G60910 | *FRUITFULL, FUL* | Chr5:24502481..24506143 reverse |
| AT1G79530 | *GAPCP1* | Chr1:29915901..29919234 reverse |
| AT4G33010 | *GLDP1* | Chr4:15926669..15931332 reverse |
| AT1G31140 | *GORDITA, AGL63, GOA* | Chr1:11117993..11119785 forward |
| AT2G22840 | *GRF1* | Chr2:9728756..9731301 forward |
| AT1G22300 | *GRF10* | Chr1:7878769..7881219 reverse |
| AT4G37840 | *HKL3* | Chr4:17790146..17792198 reverse |
| AT4G00120 | *INDEHISCENT, IND* | Chr4:41664..43197 reverse |
| AT1G68480 | *JAG* | Chr1:25684314..25686165 reverse |
| AT1G13710 | *KLUH, CYP78A5* | Chr1:4702725..4704657 reverse |
| AT5G61850 | *LFY* | Chr5:24844295..24846933 forward |
| AT4G32551 | *LUG* | Chr4:15707481..15713662 forward |
| AT5G60200 | *MONOPTEROS* | Chr5:24240975..24242523 forward |
| AT3G57670 | *NO TRANSMITTING TRACT, NTT* | Chr3:21370903..21373446 forward |
| AT1G13400 | *NUBBIN, NUB* | Chr1:4597627..4598911 forward |
| AT1G68640 | *PERIANTHIA, PAN* | Chr1:25769575..25772543 reverse |
| AT5G03680 | *PETAL LOSS, PTL* | Chr5:957611..961047 forward |
| AT1G23190 | *PGM* | Chr1:8219868..8224469 forward |
| AT5G06070 | *RABBIT EARS, RBE* | Chr5:1828150..1829186 reverse |
| AT5G02030 | *REPLUMLESS, RPL* | Chr5:395634..399041 forward |
| AT5G60690 | *REVOLUTA, REV* | Chr5:24397022..24402195 forward |
| AT2G01570 | *RGA* | Chr2:255248..257550 reverse |
| AT4G36380 | *ROTUNDIFOLIA3, ROT3* | Chr4:17187528..17192317 reverse |
| AT2G36985 | *ROTUNDIFOLIA4, ROT4* | Chr2:15534779..15535192 reverse |
| AT3G02000 | *ROXY1* | Chr3:332275..333022 reverse |
| AT4G09960 | *SEEDSTICK, STK* | Chr4:6236374..6240932 reverse |
| AT1G43850 | *SEUSS* | Chr1:16617152..16622049 forward |
| AT3G58780 | *SHATTERPROOF 1, SHP1* | Chr3:21738460..21742517 forward |
| AT2G42830 | *SHATTERPROOF 2, SHP2* | Chr2:17820255..17824013 forward |
| AT4G13890 | *SHM5* | Chr4:8031971..8033723 reverse |
| AT1G22020 | *SHM6* | Chr1:7754320..7757383 forward |
| AT1G36370 | *SHM7* | Chr1:13696041..13698639 reverse |
| AT1G15360 | *SHN1* | Chr1:5283538..5284673 forward |
| AT4G36930 | *SPATULA, SPT* | Chr4:17414127..17416294 forward |
| AT4G16340 | *SPK1, SPIKE1* | Chr4:9228185..9241317 reverse |
| AT4G37930 | *STM* | Chr4:17831731..17834927 reverse |
| AT4G17810 | *SUPERMAN, SUP* | Chr4:9906848..9907657 forward |
| AT3G15030 | *TCP4* | Chr3:5061681..5064115 forward |
| AT5G60970 | *TCP5* | Chr5:24535570..24537047 reverse |
| AT2G31070 | *TCP10* | Chr2:13220478..13222609 reverse |
| AT5G48375 | *TGG3* | Chr5:19601303..19603883 reverse |
| AT1G50030 | *TOR* | Chr1:18522441..18539995 reverse |
| AT3G09790 | *UBQ8* | Chr3:3003861..3006192 reverse |
| AT5G37640 | *UBQ9* | Chr5:14952781..14953750 reverse |
| AT4G05320 | *UBQ10* | Chr4:2718169..2720308 forward |
| AT4G05050 | *UBQ11* | Chr4:2588003..2589386 reverse |
| AT1G30950 | *UFO* | Chr1:11036180..11037508 forward |
| AT4G28190 | *ULT1* | Chr4:13985178..13987442 forward |
| AT2G20825 | *ULT2* | Chr2:8965556..8967243 reverse |
| AT5G40280 | *WIG* | Chr5:16101391..16104961 forward |
| AT1G80600 | *WIN1* | Chr1:30298499..30300553 reverse |
| AT2G28610 | *WUSCHEL, WUS* | Chr2:12262013..12263415 forward |
| AT1G08465 | *YAB2* | Chr1:2675813..2679781 forward |
| AT5G57360 | *ZTL* | Chr5:23241426..23244590 forward |

**Abraham MC, Metheetrairut C, Irish VF. 2013.** Natural variation identifies multiple loci controlling petal shape and size in *Arabidopsis thaliana*. *PLOS ONE* **8**: e56743. doi: 10.1371/journal.pone.0056743

**Anastasiou E, Kenz S, Gerstung M*, et al.* 2007.** Control of plant organ size by *KLUH*/*CYP78A5*-dependent intercellular signaling. *Developmental Cell* **13**: 843-856.

**Chen D, Yan W, Fu L-Y, Kaufmann K. 2018.** Architecture of gene regulatory networks controlling flower development in *Arabidopsis thaliana*. *Nature Communications* **9**: 4534. doi: 10.1038/s41467-018-06772-3

**Di Marzo M, Herrera-Ubaldo H, Caporali E*, et al.* 2020.** SEEDSTICK controls *Arabidopsis* fruit size by regulating cytokinin levels and *FRUITFULL*. *Cell Reports* **30**: 2846-2857.

**Dinneny JR, Yanofsky MF. 2005.** Drawing lines and borders: how the dehiscent fruit of *Arabidopsis* is patterned. *BioEssays* **27**: 42-49.

**El-Assal SED, Alonso-Blanco C, Hanhart CJ, Koornneef M. 2004.** Pleiotropic effects of the *Arabidopsis* cryptochrome 2 allelic variation underlie fruit trait-related QTL. *Plant Biology* **6**: 370-374.

**Groszmann M, Paicu T, Smyth DR. 2008.** Functional domains of SPATULA, a bHLH transcription factor involved in carpel and fruit development in *Arabidopsis*. *The Plant Journal* **55**: 40-52.

**Huang T, Irish VF. 2015.** Gene networks controlling petal organogenesis. *Journal of Experimental Botany* **67**: 61-68.

**Ma Y, Miotk A, Šutiković Z*, et al.* 2019.** WUSCHEL acts as an auxin response rheostat to maintain apical stem cells in *Arabidopsis*. *Nature Communications* **10**: 5093. doi: 10.1038/s41467-019-13074-9

**Prasad K, Zhang X, Tobón E, Ambrose BA. 2010.** The *Arabidopsis* B-sister MADS-box protein, GORDITA, represses fruit growth and contributes to integument development. *The Plant Journal* **62**: 203-214.

**Ren H, Dang X, Yang Y*, et al.* 2016.** SPIKE1 activates ROP GTPase to modulate petal growth and shape. *Plant Physiology* **172**: 358-371.

**Ripoll JJ, Zhu M, Brocke S*, et al.* 2019.** Growth dynamics of the *Arabidopsis* fruit is mediated by cell expansion. *Proceedings of the National Academy of Sciences* **116**: 25333-25342.

**Rodríguez-Hernández AA, Muro-Medina CV, Ramírez-Alonso JI, Jiménez-Bremont JF. 2017.** Modification of *AtGRDP1* gene expression affects silique and seed development in *Arabidopsis thaliana*. *Biochemical and Biophysical Research Communications* **486**: 252-256.

**Wang Q, Huang W, Jiang Q*, et al.* 2013.** Lower levels of expression of *FATA2* gene promote longer siliques with modified seed oil content in *Arabidopsis thaliana*. *Plant Molecular Biology Reporter* **31**: 1368-1375.

**Yadava SK, Paritosh K, Panjabi-Massand P*, et al.* 2014.** Tetralocular ovary and high silique width in yellow sarson lines of *Brassica rapa* (subspecies *trilocularis*) are due to a mutation in Bra034340 gene, a homologue of *CLAVATA3* in *Arabidopsis*. *Theoretical and Applied Genetics* **127**: 2359-2369.

**Zhang Y, Dai L, Liu Y, Zhang Y, Wang S. 2017.** Identifying novel fruit-related genes in *Arabidopsis thaliana* based on the random walk with restart algorithm. *PLOS ONE* 12: e0177017. doi: 10.1371/journal.pone.0177017

**Zhu L, Zhang Y-H, Su F, Chen L, Huang T, Cai Y-D. 2016.** A shortest-path-based method for the analysis and prediction of fruit-related genes in *Arabidopsis thaliana*. *PLOS ONE* **11**: e0159519. doi: 10.1371/journal.pone.0159519
